# Roles of endoplasmic reticulum stress-mediated apoptosis in M1-polarized macrophages during mycobacterial infections

**DOI:** 10.1038/srep37211

**Published:** 2016-11-15

**Authors:** Yun-Ji Lim, Min-Hee Yi, Ji-Ae Choi, Junghwan Lee, Ji-Ye Han, Sung-Hee Jo, Sung-Man Oh, Hyun Jin Cho, Dong Woon Kim, Min-Woong Kang, Chang-Hwa Song

**Affiliations:** 1 Department of Microbiology, College of Medicine, Chungnam National University, 266 Munhwa-ro, Jung-gu, Daejeon 301-747, South Korea; 2 Department of Medical Science, College of Medicine, Chungnam National University, 266 Munhwa-ro, Jung-gu, Daejeon 301-747, South Korea; 3 Department of Anatomy, College of Medicine, Chungnam National University, 266 Munhwa-ro, Jung-gu, Daejeon 301-747, South Korea; 4 Department of Thoracic and Cardiovascular Surgery, Chungnam National University Hospital, 282 Munhwa-ro, Jung-gu, Daejeon, 301-721, South Korea; 5 Research Institute for Medical Sciences, College of Medicine, Chungnam National University, 266 Munhwa-ro, Jung-gu, Daejeon 301-747, South Korea; 6 Brain Research Institute, College of Medicine, Chungnam National University, 266 Munhwa-ro, Jung-gu, Daejeon 301-747, South Korea

## Abstract

Alteration of macrophage function has an important regulatory impact on the survival of intracellular mycobacteria. We found that macrophages infected with attenuated *Mycobacterium tuberculosis* (Mtb) strain H37Ra had elevated expression of M1-related molecules, whereas the M2 phenotype was dominant in macrophages infected with virulent Mtb H37Rv. Further, the TLR signalling pathway played an important role in modulating macrophage polarization against Mtb infection. Interestingly, endoplasmic reticulum (ER) stress was significantly increased in M1 polarized macrophages and these macrophages effectively removed intracellular Mtb, indicating that ER stress may be an important component of the host immune response to Mtb in M1 macrophages. This improved understanding of the mechanisms that regulate macrophage polarization could provide new therapeutic strategies for tuberculosis.

Tuberculosis (TB), caused by *Mycobacterium tuberculosis* (Mtb), remains a global health problem. Researchers have tried to develop methods to understand the pathogenesis of TB but the exact mechanisms are still unknown. In the lung, alveolar macrophages phagocytose the infectious Mtb which are inhaled as droplets from air and kill the infectious Mtb by reactive oxygen species in the phagolysosome, and lead to recruitment of mononuclear cells^1^. In this way, macrophages play an important role in eliminating Mtb. Macrophages are the first line of immune cell defence to encounter and eradicate mycobacteria^2^. Classically activated (M1) macrophages are known to accelerate inflammatory responses and mediate resistance against intracellular parasites^3^. In contrast, alternatively activated M2 macrophages are involved in tissue repair, tumour progression, restraint of inflammation, and promotion of Th2 responses^3^. Macrophage polarization has been shown to have a critical role in disease progression and resolution of inflammatory or infectious processes^4^,^5^,^6^. In fact, macrophage polarization has been shown to modulate anti-microbial activity and cytokine production during TB granuloma formation^7^,^8^. Thus, modulation of macrophage polarization might play an important role in Mtb infection^9^,^10^.

Apoptotic cell death is an important host defence mechanism against Mtb infection, but the underlying molecular mechanisms are not clear. Apoptosis can be triggered by intrinsic and extrinsic signals, and macrophages can be activated in response to such stimuli during Mtb infection. Several publications suggest that macrophages infected with virulent Mtb show less apoptosis than those infected with attenuated Mtb^1^,^11^. Thus, we analysed the effects of macrophage polarization on apoptosis during Mtb infection.

The endoplasmic reticulum (ER) is a type of membrane-bound organelle that functions in lipid metabolism, secretory and membrane protein folding, and calcium homeostasis. Accumulation of unfolded proteins, hypoxia, glucose deprivation, oxidative stress, and bacterial infection has all been shown to induce ER stress and activate the unfolded protein response (UPR). For example, *Pseudomonas aeruginosa* infection has been shown to induce ER stress in primary bronchial epithelial cells^12^. *Brucella melitensis* infection induced UPR to promote bacterial intracellular growth^13^, and *Simkania negevensis* infection inhibited UPR to promote its intracellular proliferation^14^. Similarly, we previously showed that the Mtb ESAT-6 protein induced ER stress by disrupting calcium homeostasis in human lung epithelial cells^15^. Many reports indicate that ER stress invokes innate immune responses and is a key mediator of inflammatory signalling in response to various diseases and bacterial infections^16^,^17^. However, it is not clear whether induction of UPR or ER stress benefits the host or the pathogenic bacteria. Our previous findings suggest that ER stress-mediated apoptosis plays a critical role in regulating intracellular mycobacteria^18^,^19^. Therefore, regulation of ER stress might be a novel way to suppress intracellular mycobacteria in macrophages.

Here we show that M1-polarized macrophages can more effectively remove mycobacteria by upregulating ER stress responses than can M2-polarized macrophages. Based on these results, we propose the eradication of intracellular mycobacteria as a novel role for ER stress-mediated apoptosis in M1-polarized macrophages. These observations support the idea that macrophage polarization is important for host defence in mycobacterial infection, and results from our study might contribute to improved therapeutic strategies to remove Mtb.

## Results

### Mtb infection may regulate characterization of macrophages

**Figure 1:**
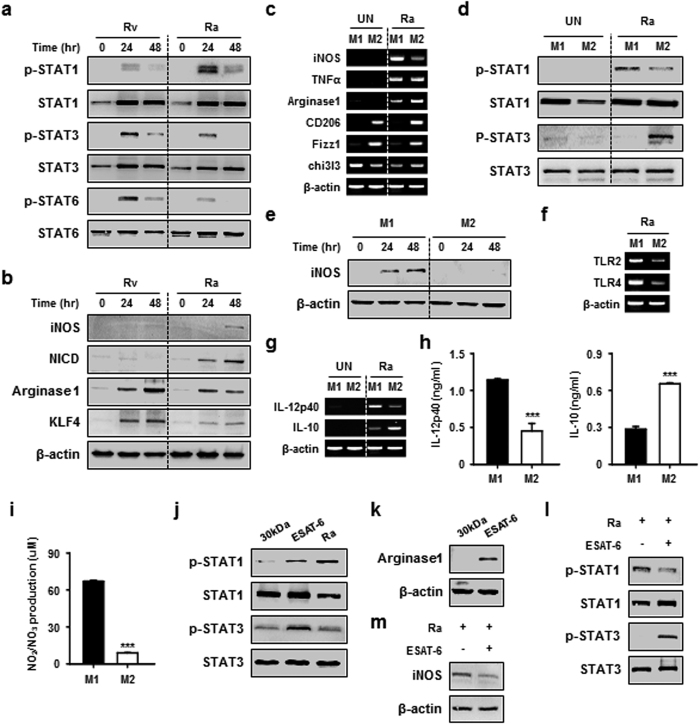
Mtb Infection regulates macrophage polarization. (**a**,**b**) BMDMs (M0) were infected with either H37Rv (Rv) or H37Ra (Ra) cells at an MOI = 1 for the indicated times. After infections, (**a**) STATs, and (**b**) iNOS, NICD, Arginase 1 and KLF4 were detected using western blot. Total STATs and β-actin were used for cell loading controls. (**c**) BMDMs were polarized into M1 and M2 phenotypes, as described in Figure S1, and then M1 or M2 macrophages were infected with H37Ra for 24 h. mRNA levels of M1/M2-associated markers were evaluated using quantitative PCR. (**d**,**e**) M1 and M2 macrophages were infected with H37Ra for 24 h. Cell lysates were analysed for (**d**) STAT1, p-STAT1, STAT3, and p-STAT3, and (e) iNOS expression was analysed by western blot. Untreated cells (UN) were used as a control. (**f**,**g**) M1 or M2 macrophages were infected with H37Ra for 24 h. mRNA levels for (**f**) TLR2 and TLR4, and (**g**) IL-12p40 and IL-10, were measured using quantitative PCR. Untreated cells (UN) were used as a control. (**h**,**i**) Using supernatant fractions from cell cultures, (**h**) IL-12p40 and IL-10 cytokine secretions, and (**i**) NO production were measured. (**j**,**k**) BMDMs (M0) were stimulated with 30 kDa (10 μg/ml) and ESAT-6 (10 μg/ml) antigens for 24 h, and assessed for (**j**) phosphorylation of STAT1, p-STAT1, STAT3, and p-STAT3, and (**k**) Arginase-1 expression. (**l**,**m**) BMDMs (M0) were infected with H37Ra, medium was changed, and then cells were treated with ESAT-6. Treated cells were incubated for 24 h, and then confirmed for the expression of (**l**) STAT1 and STAT3, and (**m**) iNOS. For all panels, results are representative of at least three experiments. ***denotes statistically significant differences with a *p*-value of <0.001.

To investigate changes in macrophage phenotype that result from mycobacterial infection, bone marrow derived macrophages (BMDMs) were infected either with virulent Mtb H37Rv or attenuated Mtb H37Ra at a multiplicity of infection (MOI) of 1, and macrophage phenotypes were monitored. We found more M2-related molecules (e.g. STAT3, STAT6, arginase 1, and KLF4) in H37Rv-infected macrophages than in H37Ra-infected cells. In contrast, more M1-related molecules (e.g. STAT1, iNOS, and NICD) were seen in H37Ra-infected macrophages than in H37Rv-infected macrophages (Fig. 1a,b). Macrophages appear to flexibly adapt to strains of mycobacteria, and Mtb virulence appears to be a key determinant of mycobacterial survival and replication in host macrophages.

To explore the function of polarized macrophages during mycobacterial infections, we prepared M1- or M2-polarized macrophages *in vitro* (Figure S1), infected them with Mtb H37Ra, and analysed the activation of M1- or M2-related molecules. The mRNA expression profiles in these macrophages correlated with western blot analysis results in Ra-infected macrophages (Fig. 1c). M1-related p-STAT1 was induced in Mtb H37Ra-infected M2-polarized macrophages (Fig. 1d). Interestingly, Mtb H37Ra-infected M1-polarized macrophages induced iNOS production in a time-dependent manner (Fig. 1e). Next, we asked whether TLR signalling is involved in macrophage polarization during mycobacterial infection. Increased expression of TLR2 and TLR4 was observed in H37Ra-infected M1-polarized macrophages (Fig. 1f). We found that the level of IL-12 expression (Fig. 1g,h) and NO production (Fig. 1i) were increased in M1-polarized macrophages after H37Ra infection. These observations were confirmed using Raw264.7 cells (Figure S2).

We sought to determine, which mycobacterial factors are involved in macrophage polarization. Mycobacterial ESAT-6, an Mtb virulence factor, strongly induced M2-related molecules (e.g. p-STAT3 and arginase1) (Fig. 1j,k), suggesting that ESAT-6 can skew macrophage function toward the M2 phenotype during mycobacterial infection. ESAT-6 treatment shifted H37Ra-infected macrophages from an M1 to M2 phenotype. We confirmed this observation using a combination of ESAT-6 treatment and H37Ra infection. The combination of these two factors induced M2-like types in M1-polarized macrophages by H37Ra infection, as indicated by increase STAT3 phosphorylation and decrease in STAT1 and iNOS activation (Fig. 1l,m). These data suggest that virulent mycobacteria can skew the macrophage function toward the M2 phenotype, particularly through the production of ESAT-6, to manipulate the environment for survival in the host.

### TLR signalling is essential to modulate Mtb-induced macrophage polarization

**Figure 2:**
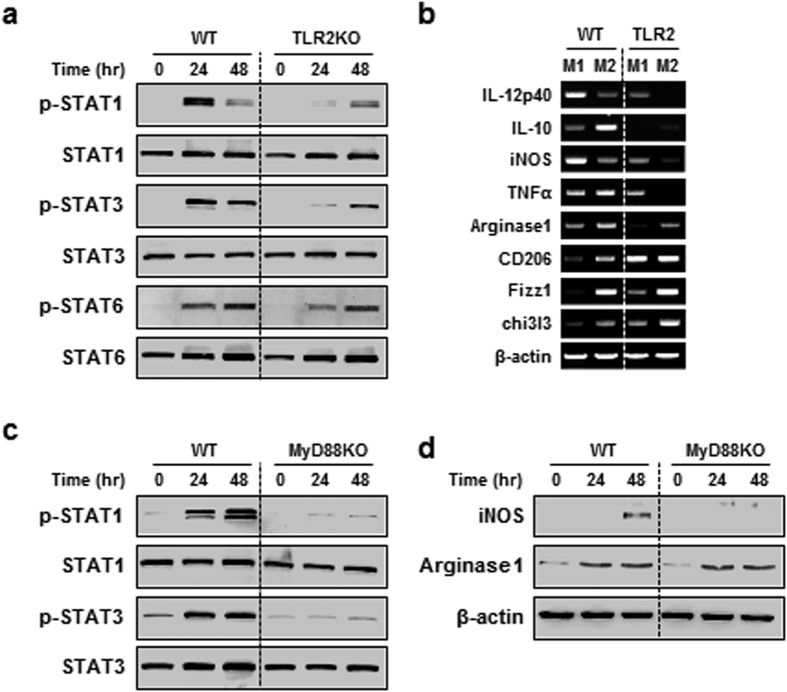
TLR2/MyD88 signalling is essential for Mtb-modulated macrophage polarization. (**a**) BMDMs (M0) from wild-type (WT) and TLR2-deficicent mice were infected with H37Ra. After harvesting, the cells were analysed for the expression of phosphorylated and total STATs by western blot. (**b**) WT and TLR2-deficient BMDMs were polarized to exhibit M1 and M2 phenotypes, and then they were infected with H37Ra for 24 h. These cells were analysed for mRNA expression of M1/M2-related genes using quantitative PCR. (**c**,**d**) BMDMs (M0) from WT and MyD88-deficient mice were infected with H37Ra and analysed by western blot for (**c**) p-STAT1 and p-STAT3, and (**d**) iNOS and arginase 1. For all panels, the results are representative of at least three independent experiments.

A recent study suggests that Toll-like receptor (TLR) pathway is important to induce macrophage polarization during *Vibrio cholera* infection^20^. To address whether TLR signalling regulates macrophages during mycobacterial infection, we evaluated the expression of M1 and M2 markers through the H37Ra-activated TLR signalling, using BMDMs of wild-type, TLR2 and TLR4 deficient mice (Figure S3a). TLR2-deficient macrophages exhibited decreased M1 phenotype marker, p-STAT1 (Fig. 2a). In contrast, no significant differences in macrophages from TLR4 KO mice were observed between the wild-types (Figure S3b,c). These results were confirmed with low levels of M1 marker gene mRNA expression in TLR2-deficient macrophages (Fig. 2b). As TLR2 signalling is MyD88-dependent pathway, we hypothesized that MyD88 molecule also influences macrophage phenotypes into M1 polarization. As expected, M1 macrophage phenotype markers also decreased in MyD88-deficient BMDMs (Figure S3d) during H37Ra infections in macrophage-polarized condition (Fig. 2c,d). Although some M2 markers such as IL-10 or p-STAT3 were weakly induced in TLR2 KO or MyD88 KO BMDMs, most of M2 markers were strongly expressed in TLR2 KO or MyD88 KO BMDMs. These results indicate that TLR2/MyD88-dependent signalling is essential for induction of M1 macrophage polarization during H37Ra infection.

### Mtb-mediated M1 macrophage apoptosis is associated with ER stress response

**Figure 3:**
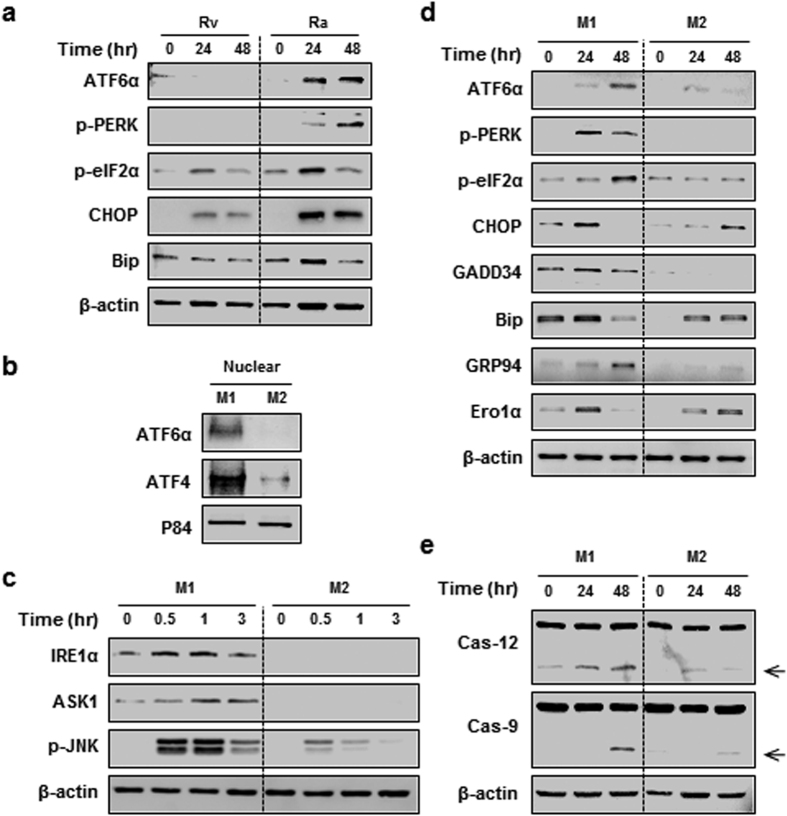
Mtb-mediated M1 macrophages are associated with ER stress. (**a**) BMDMs (M0 macrophages) were infected with H37Rv and H37Ra for the indicated times, and analysed for expression proteins associated with the ER stress response. (**b**) After H37Ra infection for 24 h, nuclear fractions were isolated from macrophages, and analysed for the active forms of ATF6 and ATF4 by western blot. p84 was used as a loading control for nuclear extracts. (**c**,**d**) M1 and M2 macrophages were infected with H37Ra for the indicated times, and analysed for expression proteins associated with the ER stress response, including (**c**) members of the IRE1α signalling pathway, and (**d**) ATF6α and p-PERK signalling pathway. (**e**) Using western blot analysis, activation of the ER stress-mediated caspases, Cas-12 and Cas-9, was measured in M1- and M2-conditioned BMDMs following H37Ra infection for the indicated times.

In our previous reports, we suggested that ER stress-mediated apoptosis plays an important role in the regulation of mycobacterial infection^18^,^21^. We next determined whether virulent mycobacteria could modulate macrophage polarization to control ER stress. Interestingly, attenuated H37Ra-infected M0 macrophages exhibited an increase in the abundance of several ER-stress-associated molecules, whereas the virulent H37Rv strain did not have this effect (Fig. 3a). To clarify whether the magnitude of ER stress is affected by macrophage polarization, we measured the expression levels of ER stress-associated molecules in M1 and M2 macrophages after mycobacterial infection. The expression of ATF6α, IRE1α, p-PERK, p-eIF2α, CHOP, GADD34, Bip, GRP94, and Ero1α was significantly induced in M1 macrophages, suggesting that M1 macrophages could induce ER stress in a manner that is favourable for controlling intracellular mycobacteria (Fig. 3b–d). Mycobacterial-infected macrophages exhibited a much higher level of caspase activation in M1 macrophages than in M2 macrophages (Fig. 3e).

**Figure 4:**
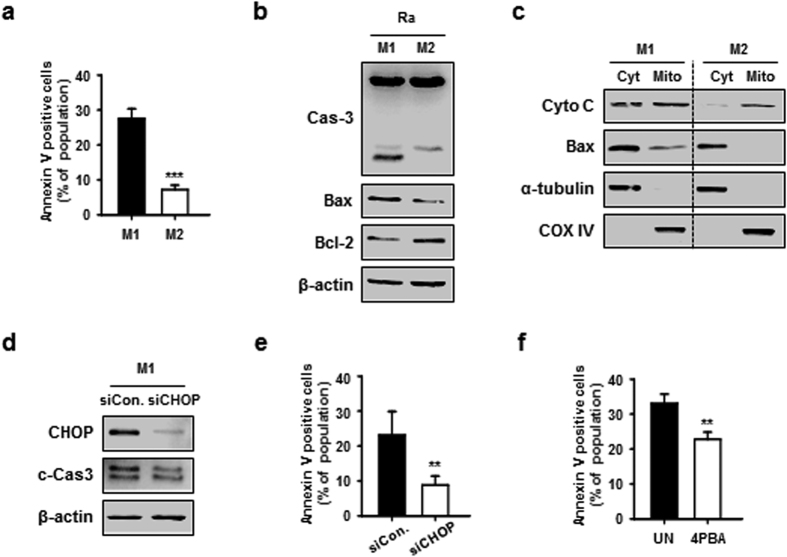
ER stress-mediated apoptotsis is strongly induced in Mtb-infected M1 macrophages. (**a**) M1 and M2 macrophages were infected with H37Ra for 24 h, and levels of apoptosis were measured by Annexin-V/PI staining. Bar graphs depict the percentage of Annexin-V positive/PI negative cells. (**b**) M1 and M2 macrophages infected with H37Ra for 24 h. Caspase-3 activation and Bax/Bcl-2 expression were measured by western blot. (**c**) Experiments were conducted as in (**b**), but H37Ra-infected cell lysates were separated into cytosolic and mitochondrial fractions. The fractions were immunoblotted for cytochrome C and Bax. For loading controls, α-tubulin (cytosolic) and COX IV (mitochondrial) were used. (**d**,**e**) M1 macrophages were transfected with CHOP siRNA (200 nM) for 24 h, and then infected with H37Ra for the indicated times, and analysed for (**d**) activation of CHOP and caspase-3 by western blot and (**e**) apoptosis by Annexin-V/PI staining. Bar graphs depict the percentage of Annexin-V positive/PI negative cells. (**f**) M1 macrophages were pre-treated with ER stress inhibitor (4PBA, 5 μM) for 2 h, and then infected with H37Ra for 24 h. Apoptosis was measured by Annexin-V/PI staining. For all panels, the results are representative of three independent experiments; *denotes statistically significant differences with a *p*-value of <0.05; **denotes statistically significant differences with a *p*-value of <0.01.

To investigate the role of macrophage polarization during mycobacterial infection, we analysed the apoptotic effects induced by H37Ra infection. Interestingly, M1 macrophages showed significantly higher percentage of cells undergoing apoptotic cell death than did M2 macrophages during Mtb infection (Fig. 4a). Activation of caspase-3, Bax, and cytochrome C was increased in H37Ra-infected M1 macrophages compared to M2 macrophages (Fig. 4b,c). CHOP knockdown and 4-phenylbutyrate (4PBA) pre-treatment decreased H37Ra-induced CHOP expression and apoptosis in M1 macrophages (Fig. 4d–f). These results show that ER stress-mediated apoptosis is more associated with M1-polarized macrophages than M2 macrophages during Mtb infection.

### The relationship between macrophage polarization and intracellular mycobacterial survival

**Figure 5:**
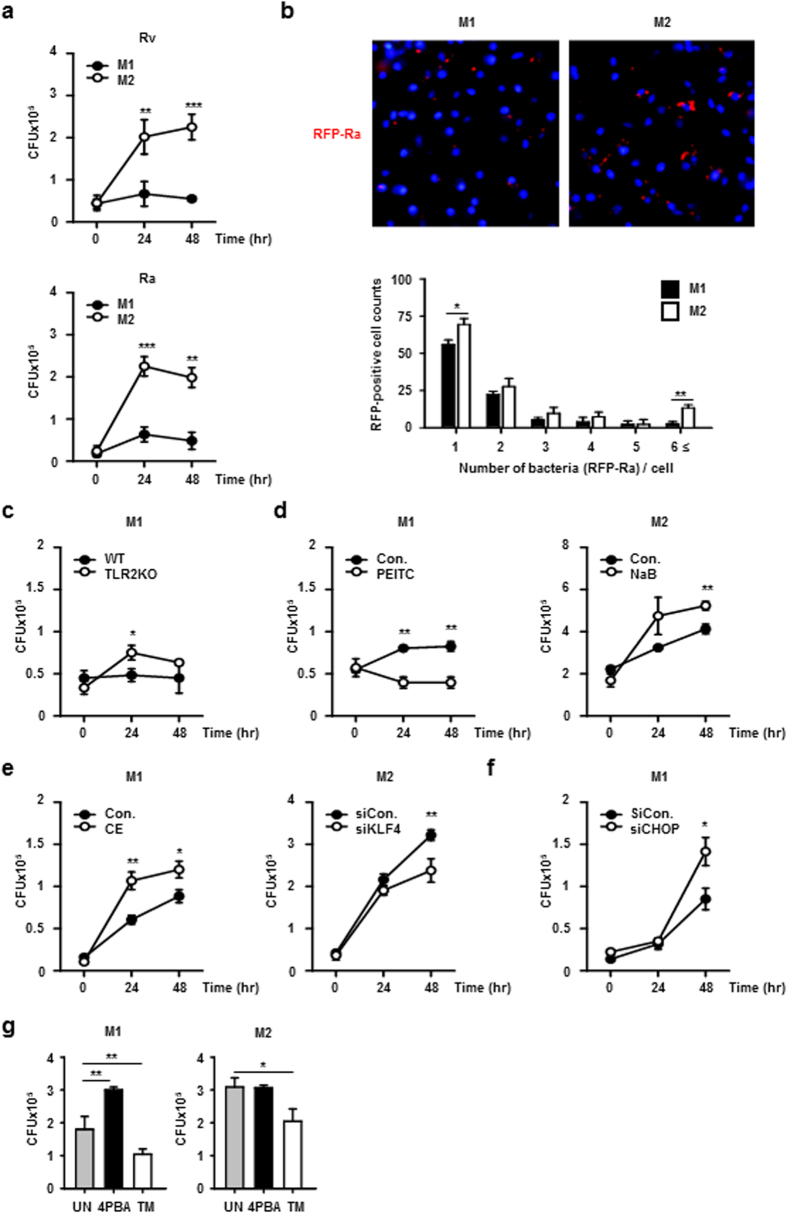
Macrophage polarization mediates intracellular mycobacterial survival. (**a**) M1 and M2 macrophages were infected with either H37Rv (top, Rv) or H37Ra (bottom, Ra). After 48 h of incubation, intracellular Mtb survival was determined by CFU enumeration. (**b**) Top: M1 or M2 macrophages were infected with RFP-labelled H37Ra for 48 h, and the infection was detected by fluorescence microscopy. Nuclei were stained with DAPI (blue fluorescence). Bottom, numbers of RFP-H37Ra per infected cell were counted manually. (**c**) WT and TLR2-deficient BMDMs were polarized to exhibit M1 and M2 phenotypes. M1 and M2 macrophages were infected with H37Ra, and intracellular survival of H37Ra was measured by CFU enumeration. (**d**) M1 and M2 macrophages were pre-treated with inducers for 2 h as follows: M1 macrophages received NICD inducer (PEITC, 2.5 μM) and M2 macrophages received KLF4 inducer (NaB, 1 mM). M1 and M2 cells were then infected with H37Ra for 24 or 48 h. Intracellular survival of H37Ra was measured by CFU enumeration. (**e**) M1 macrophages were pre-treated with γ-secretase inhibitor (compound E, 5 μM) for 2 h. M2 macrophages were transfected with KLF4 siRNA for 24 h. Then, M1 and M2 cells were infected with H37Ra for the indicated times and analysed for the intracellular survival of Mtb by CFU enumeration. (**f**) M1 macrophages were transfected with CHOP siRNA for 24 h, and then infected with H37Ra for the indicated times and analysed for the intracellular Mtb by CFU enumeration. (**g**) M1 and M2 macrophages were pre-treated with ER stress inhibitor (4PBA, 5 μM) or inducer (tunicamycin; TM, 500 ng/ml) for 2 h prior to H37Ra infection. After incubation for 48 h, the intracellular survival of H37Ra was measured by CFU. For all panels, the results are representative of three independent experiments; *denotes statistically significant differences with a *p*-value of <0.05; **denotes statistically significant differences with a *p*-value of <0.01.

To determine the role of macrophage polarization in mycobacterial infection, we analysed the survival of Mtb in M1 and M2 macrophages. In M1 macrophages, intracellular Mtb replication was significantly inhibited compared to that in M2 macrophages (Fig. 5a). Similarly, confocal microscopy showed that intracellular survival of H37Ra was more effective in M2 macrophages than in M1 cells (Fig. 5b). Based on our findings shown in Fig. 2, it was possible that TLR signalling is important to the signalling that leads to the M1 phenotype. Therefore, we assessed the survival of mycobacteria in M1-polarized TLR2-deficient macrophages. H37Ra bacteria survived more effectively in TLR2-deficient M1 macrophages than in wild-type M1 macrophages (Fig. 5c). Based on our observations of NOTCH intracellular domain (NICD [M1-related response]) and Krüppel-like factor 4 (KLF4 [M2-related response]) shown in Fig. 1b, we examined the regulatory effects of NICD or KLF4 on intracellular survival of mycobacteria in M1 and M2 macrophages. Pre-treatment of M1 macrophages with the NICD inducer, PEITC, caused a small but significant decrease in intracellular bacterial survival (Fig. 5d). Conversely, pre-treatment of M2 cells with the KLF4 inducer, NaB, led to an increase in intracellular bacterial survival. Finally, intracellular survival of H37Rv was significantly increased in M1 macrophages pre-treated with the γ-secretase inhibitor, compound E, compared to the untreated control cells (Fig. 5e). The intracellular survival ratio in KLF4 knockdown cells was slightly (but significantly) decreased. Similarly, NICD inhibition in M1 macrophages or KLF4 induction in M2 macrophages increased intracellular survival of Mtb H37Rv. In contrast, NICD activation in M1 cells or KLF4 knockdown in M2 macrophages significantly suppressed H37Rv survival (Figure S4). To identify the biological roles of ER stress during mycobacterial infection, M1 and M2 macrophages were transfected with siCHOP, or pre-treated with 4PBA and tunicamycin (TM) prior to infection. CHOP knockdown in M1 macrophage induced Mtb survival (Fig. 5f). Pre-treatment with 4PBA increased the intracellular survival of H37Ra in M1 macrophages, but not in M2 macrophages, suggesting that M1-polarized cells are closely associated with ER stress to regulate intracellular mycobacteria (Fig. 5g). Contrastively, pre-treatment of tunicamycin decreased the Mtb survival in M2 macrophages.

**Figure 6:**
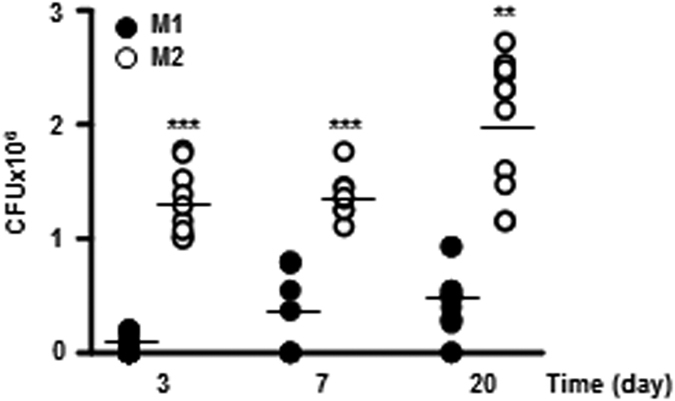
Intracellular survival of Mtb H37Ra in mouse models of M1- and M2-like polarization. Mouse models of M1- and M2-like polarization were developed by nasal injection of the following inducers: LPS and IFN-γ for M1-like polarization, IL-4 and IL-13 for M2-like polarization. These M1- and M2-like mouse models were infected intranasally with H37Ra (5 × 10^6^) for 3, 7, or 20 days, and then the mycobacterial burden was determined in the lungs of these mice by CFU enumeration. The data reflect nine to 12 mice per time point for each group, and are representative of three independent experiments. For all panels, *denotes a *p*-value of <0.05, **denotes a *p*-value of <0.01, and ***denotes a *p*-value of <0.001.

Next, we investigated the effect of polarization on the intracellular survival of mycobacteria using *in vivo* models, forcing M1 or M2 activation in the lungs of wild-type C57BL/6 mice (Figure S5). The levels of intracellular survival of mycobacteria in mice with M2 polarized inflammatory cells were higher than those in mice with M1 polarized inflammatory cells (Fig. 6). This result indicates that M1 macrophage polarization contributes to the elimination of intracellular mycobacteria.

## Discussion

Macrophages are the first immune cells to encounter Mtb cells after infection. The functional phenotypes of macrophages may be classified as M1 or M2. During Mtb infection, apoptosis and activation of macrophages are important events that reduce the number of intracellular Mtb^1^,^18^,^22^. Although activated macrophages play critical roles in host defence against mycobacterial infection, the function of M1- or M2-polarized macrophages during TB infection has not been clearly elucidated. In this study, we demonstrate that M1 macrophages are activated by an H37Ra infection, but M2 macrophage development is promoted by an H37Rv infection. The major difference between H37Rv and H37Ra is a mutation in *phoP*, a transcriptional regulator that leads to the secretion of antigens such as ESAT-6^23^. ESAT-6 is a known, immunodominant antigen of Mtb, it downregulates IRFs and modulate host immune signalling^24^. It would be required for mycobacterial survival in the host. Here, we show that ESAT-6 is one of the candidate mycobacterial antigens associated with M2 macrophage polarization. The combination effect of H37Ra infection and ESAT-6 stimulation implies that ESAT-6 has the capacity to shift macrophages toward the M2 phenotype. These results suggest that Mtb interrupts M1 macrophage polarization by secreting virulence factors such as ESAT-6 that block the M1-associated factors^24^.

It is well established that M1 polarized macrophages trigger the fusion of lysosomes with Mtb-containing phagosomes and induce iNOS upregulation^5^. M1 macrophages are linked to the upregulation of microbicidal enzymes and inducible nitric oxide generation. We observed that M1 macrophage polarization is important for the elimination of intracellular mycobacteria. Importantly, M1 polarization also ensures that the TLR2 signalling pathway is utilized over the TLR4 signalling pathway. Others have suggested that Mtb inhibits MyD88-dependent TLR2 signalling^24^. Our results suggest that the TLR2/MyD88 signalling pathway is relatively more associated with M1 macrophage activation than M2 polarization during Mtb infection. Therefore, M1 macrophage activation via the TLR2 signalling pathway could be beneficial to the host by inhibiting the intracellular survival of Mtb.

Previously, we suggested that ER stress-mediated apoptosis is important for the regulation of intracellular survival of mycobacteria^15^,^18^,^21^. We noted here that the ER stress response is increased in M1 macrophages after Mtb infection. One of the typical M1 macrophage characteristics is high production of nitric oxide (NO) and reactive oxygen intermediates (ROI)^25^. NO and ROI production can induce the ER stress response^26^. It is well known that classically activated macrophages (M1) secrete proinflammatory cytokines, such as TNF-α and IL-6^5^. Induction of TNF-α and IL-6 is associated with the induction of ER stress pathway^27^. Our results suggest that M1 macrophages facilitate ER stress during Mtb infection. Level of phospho-eIF2α protein was definitely higher in Ra-infected cells than Rv (Fig. 3a). It seems that this mechanism would be appropriate for regulating host immune responses during mycobacterial infection. Previously we showed that virulent mycobacteria (Mtb H37Rv) might inhibit eIF2α phosphorylation to regulate the UPR for evading antimycobacterial cellular immune responses in macrophages. In addition to ER stress, the integrated stress response such as general control non-derepressible 2 (GCN2), haem-regulated inhibitor (HRI) or protein kinase double stranded RNA dependent (PKR) can be activated during mycobacterial infection^28^. A previous report suggests that ER stress in TB granulomas is associated with apoptosis as a host response to infection^29^. Therefore, we suggest that ER stress activation in M1 macrophages is important for the removal of intracellular mycobacteria in Mtb-infected macrophages. Thus, the regulatory mechanisms associated with macrophage polarization could provide new therapeutic strategies for TB.

## Experimental procedures

### Mice and cell culture macrophage differentiation and polarization

Six-to eight-week-old female wild-type and TLR4-, TLR2-, and MyD88-deficient C57BL/6 J mice were used in all experiments. BMDMs were generated by flushing bone marrow cells from femurs and tibias, and culturing for 4 days in Dulbecco’s minimal essential medium (DMEM) containing 10% fetal bovine serum (FBS), penicillin (100 IU/mL), streptomycin (100 μg/mL), 25 ng/ml granulocyte-macrophage colony-stimulating factor (GM-CSF; R&D Systems), or 25 ngml macrophage colony-stimulating factor (M-CSF; R&D Systems). The mouse monocyte/macrophage cell line Raw264.7 was maintained in DMEM containing 10% FBS, penicillin (100 IU/mL) and streptomycin (100 μgmL). M1 polarization of macrophages were induced by 10 ng/ml lipopolysaccharide (LPS; InvivoGen) and 10 ng/ml mouse IFN-γ (R&D Systems); M2 polarization was induced by 10 ng/ml mouse IL-4 (R&D Systems) and 10 ngml mouse IL-13 (R&D Systems). After 24 h, cells were infected with Mtb H37Rv or Mtb H37Ra (MOI=1), and incubated as described.

### Ethics statement concerning animal work

The authors confirm that all experiments were performed in accordance with relevant guidelines and regulations. Animal work methods were carried out in accordance with procedures that were approved by the Institutional Animal Care and Use Committee of Chungnam National University, South Korea (Permit number: CNU-00425). All animal experiments were performed in accordance with the Korean Food and Drug Administration (KFDA) guidelines.

### Bacteria, and intracellular survival analysis

Mtb H37Rv type strain (ATCC 27294) and H37Ra type strain (ATCC 25177) were obtained from the American Type Culture Collection (ATCC). Mtb used in this experiment were prepared *in vitro* as described previously^18^,^21^. Heat-killed mycobacteria were prepared by incubating H37Rv and H37Ra suspensions at 80 °C for 30 min, and stored at −80 °C.

Cells were infected *in vitro* with Mtb at an MOI of 1, and incubated for 3 h at 37 °C in 5% CO_2_. After allowing time for phagocytosis, the cells were washed three times with PBS to remove extracellular bacteria, and incubated with fresh medium without antibiotics for an additional 24 or 48 h. Cells were then lysed in sterile distilled water, and disintegrated in a water bath sonicator for 3 to 5 min to collect intracellular bacteria. The lysates were plated separately on 7H10 agar plates and incubated for 14–21 days. Colony counts were performed in triplicate.

### Preparation and purification of the mycobacterial antigens

ESAT-6 and 30 kDa antigens were purified from Mtb H37Rv using the approach of Choi HH *et al*^15^. Briefly, we used plasmid pMRLB.7 (pET23b-base construct) containing recombinant protein antigens. Recombinant proteins were expressed as NH2-terminal 6 × His-tagged fusion proteins, and purified from *Escherichia coli* cells to near homogeneity using metal chelate affinity chromatography. To remove endotoxins, these antigens were passed through a column consisting of polymyxin B sepharose gel.

### M1/M2-like mouse modelling and tissue sampling

C57BL/6 mice were treated as follows: The M1-like phenotype received a mixture of 0.5 μg LPS and 0.5 μg IFNγ in 40 μl PBS; the M2-lilke phenotype received a mixture of 0.5 μg IL-4 and 0.5 μg IL-13 in 40 μl PBS. After 24 h, mice were intranasally infected with 5 × 10^6^ H37Ra in 50 μl PBS for 3 days. Every 5~6 days for a total of three challenges, mice were intranasally treated with IFN-γ or IL-4. Lungs and blood serum were collected from individual mice on days 3, 7, and 20 after intranasal infection with H37Ra (Figure S5). Lung samples were homogenized in cold PBS and diluted to determine colony forming units (CFU), or sample lysates were analysed by western blot.

### RNA Isolation and qPCR

Total RNA was isolated from cultured macrophages or mouse tissue samples using TRIzol reagent (Invitrogen) according to the manufacturer’s instructions, and mRNA was reverse transcribed into cDNA using the Reverse Transcription kit (ELPIS Biotech). Quantitative PCR (qPCR) was performed using Prime Taq Premix (Genet Bio) to detect mRNA levels of macrophage polarization-related target genes. β-actin was used as a reference control.

### Antibodies and reagents

Cell lysates were prepared from macrophages, and western blots were performed using antibodies against p-STAT1, STAT1, p-STAT3, STAT3, STAT6, KLF4, caspase-3, Bax, Bcl-2, p-PERK, p-eIF2α, CHOP, Bip, GRP94, Ero1α, COX IV (1:1000, Cell Signaling), p-STAT6, iNOS, arginase 1, IRE1α, ASK1, ATF6α, p84 (1:1000, Santa Cruz), NICD (1:1000, Millipore), Cytochrome C (1:1000, BD Biosciences), and GADD34, ATF4, α-tubulin (1:2000, Abcam). Goat anti-rabbit IgG (1:2000, Cell Signaling) and goat anti-mouse IgG (1:2000, Calbiochem) were used as secondary antibodies. β-actin (1:5000, Santa Cruz) was used as a loading control.

Macrophages were pretreated with inhibitors or inducers for 2 h prior to Mtb infection. 4-phenylbutyrate (4PBA), protein folding chemical chaperone, reduces misfolded protein accumulation in the ER. Tunicamycin (TM), inhibitor of N-linked protein glycosylation hinders, is required for proper protein folding. Phenethyl isothiocyanate (PEITC) and sodium butyrate (NaB) were used for transcriptional activator of Notch signalling and up-regulator of KLF4 expression, respectively. All reagents were purchased from Sigma-Aldrich.

### Transfections

Transfection of siControl (200 nM, Santa Cruz), siCHOP (200 nM, Bioneer Corporation) and siKLF4 (200 nM, Santa Cruz) into the cells were performed using Lipofectamine 3000 (Invitrogen) according to the manufacturer’s instructions.

### Measurement of apoptotic cells and caspase activation

To confirm the ratio of apoptotic or necrotic cells, BMDMs were stained using an Annexin-V/PI staining kit (BD Biosciences), and analysed using a FACS Canto II flow cytometer (BD Immunocytometry Systems). Caspase activation was determined using a FLICA assay (ImmunoChemistry Technologies).

### ELISA

Secretion of IL-12p40, IL-10, TNF, IL-6, and MCP-I (BD Biosciences) was determined in medium from cultured BMDMs or in murine blood serum using a sandwich enzyme-linked immune sorbent assay (ELISA). All assays were performed according to the manufacturer’s instructions. Triplicate samples were analysed using an ELISA reader and compared to a standard curve.

### Nitric oxide assay

To measure nitric oxide (NO) production, cell culture supernatant fractions were analysed using a Griess assay. Briefly, culture medium (100 μl) was incubated with the Griess reagent (100 μl) at room temperature for 10 min, and measured by absorbance at 541 nm. Sodium nitrite was used to create a standard concentration curve.

### Subcellular fractionation

M1 and M2 macrophages were collected after infection for indicated periods. Cytosolic and mitochondrial fractions were separated using a kit (Thermo); nuclear extracts were separated using an Active Motif kit. Both protocols were conducted according to the manufacturer’s instructions. Immunoblotting antibodies against α-tubulin, COX IV, and p84 were used to check for cross-contamination and as internal controls for cytosol, mitochondria and nuclei, respectively.

### Data analysis and statistics

Data are shown as means ± SD; all experiments were performed at least three times. *In vivo* assays were done in triplicate, and a minimum of three mice was used per group. All experimental results were statistically evaluated using student’s t test or one-way analysis of variance followed by Bonferroni’s multiple comparison tests. Statistical significance between groups was determined using Mann-Whitney and Kruskal-Wallis tests. Differences were considered significant when *p-*values were <0.05 and a difference with P < 0.001 was considered highly significant.

## Additional Information

**How to cite this article**: Lim, Y.-J. *et al.* Roles of endoplasmic reticulum stress-mediated apoptosis in M1-polarized macrophages during mycobacterial infections. *Sci. Rep.* **6**, 37211; doi: 10.1038/srep37211 (2016).

**Publisher’s note:** Springer Nature remains neutral with regard to jurisdictional claims in published maps and institutional affiliations.

## Supplementary Material

Supplementary Information
