# Supplementary material for: Roles of endoplasmic reticulum stress-mediated apoptosis in M1-polarized macrophages during mycobacterial infections
Source: Sci Rep. 2016 Nov 15;6:37211. doi: 10.1038/srep37211 (PMC5109032; doi:10.1038/srep37211)
Supplement: Supplementary Information [file srep37211-s1.pdf]

Roles of endoplasmic reticulum stress-mediated apoptosis in M1-polarized macrophages during mycobacterial infections

Supplementary information

Yun-Ji Lim, Min-Hee Yi, Ji-Ae Choi, Jung-hwan Lee, Ji-Ye Han, Sung-Hee Jo, Sung-Man Oh, Hyun Jin Cho, Dong Woon Kim, Min-Woong Kang and Chang-Hwa Song

Supplementary Figure 1

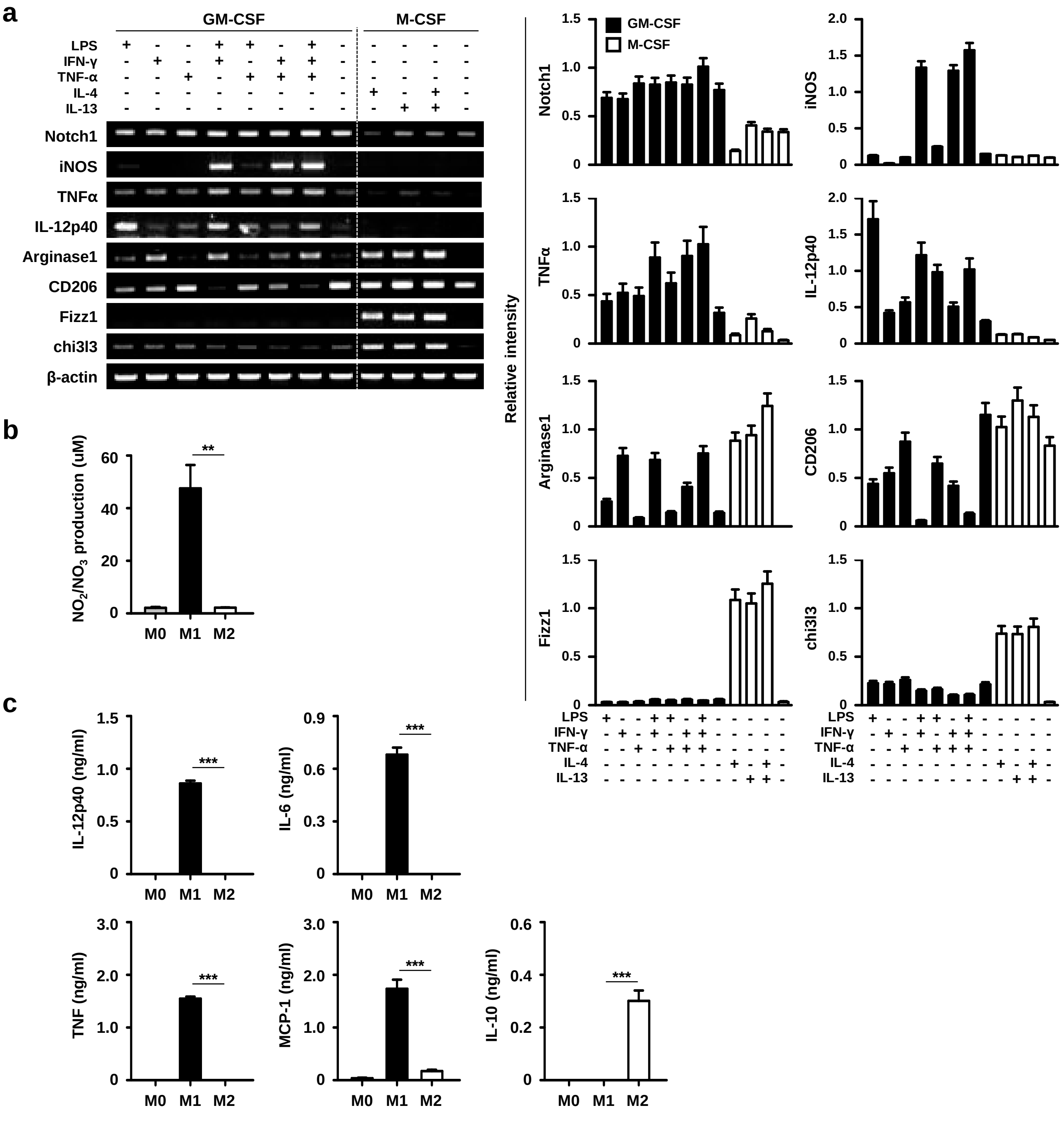

Supplementary figure 1, related to Figure 1. Cytokine-induced macrophage polarization

(a) BMDMs were treated with LPS, IFN-γ, and TNF-α (M1 stimulus) or IL-4 and IL-13 (M2 stimulus). Twenty-four hours later, macrophage mRNA was harvested and subsequently analysed for indicated macrophage polarization marker genes by qPCR *(Left).* Relative intensities of the bands analysed by quantitative densitometry are shown *(Right).*

(b and c) Using supernatant fractions from cell cultures, (b) NO production, and (c) IL-12p40, IL-6, TNF, MCP-1, and IL-10 cytokine secretions were measured.

Data are representative of three independent experiments (n=3). Statistically significant differences are indicated; *p <0.05, ** p <0.01 and *** p <0.001. M0=unstimulated macrophages.

Supplementary Figure 2

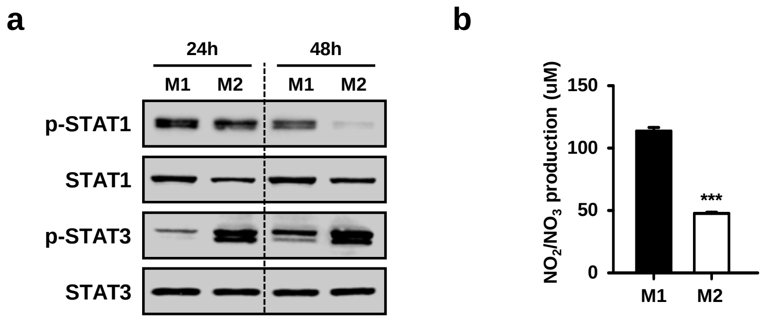

Supplementary figure 2, related to Figure 1. Increased M1-phenotype markers in H37Ra-infected Raw264.7 cells

(a) Raw264.7 cells were polarized into M1 and M2 phenotypes, as described in Figure S1, and then M1 or M2 macrophages were infected with H37Ra for 24 h. Cell lysates were analysed for STAT1, p-STAT1, STAT3, and p-STAT3 using western blot.

(b) Using supernatant fractions from cell cultures, NO production was measured.

Data are representative of three independent experiments (n=3). Statistically significant differences are indicated; *p <0.05, ** p <0.01 and *** p <0.001.

Supplementary Figure 3

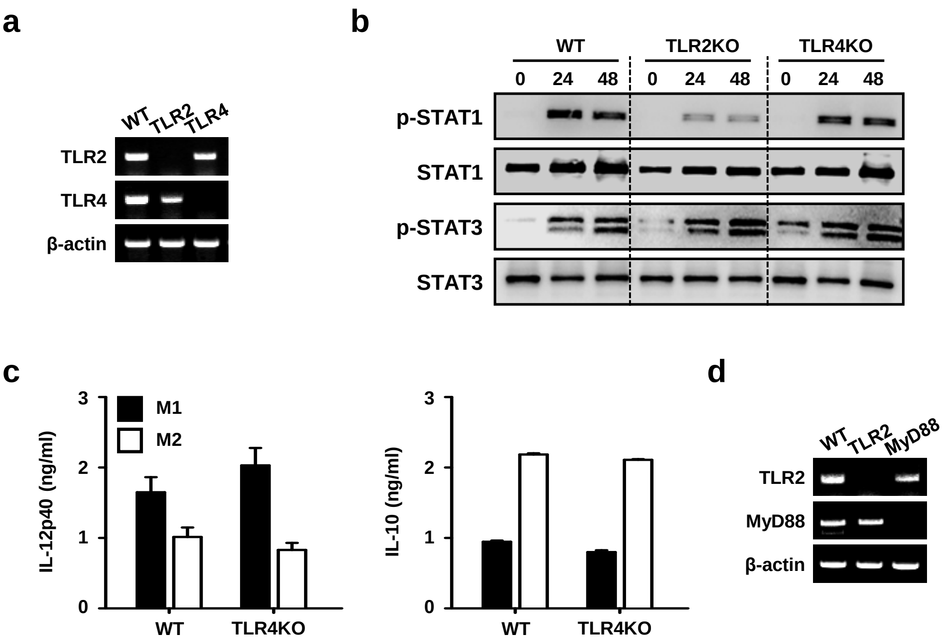

Supplementary figure 3, related to Figure 2. TLR4 Signaling is not involved in modulation of macrophage polarization during Mtb H37Ra infection

(a) Genotyping of TLR2- or TLR4-deficient mice was performed using qPCR with specific primers.

(b-c) BMDMs from WT, TLR2-, and TLR4-deficient mice were infected with H37Ra for indicated time. These cells were analysed for (b) p-STAT1 and p-STAT3 using western blot. Using supernatant fractions from cell cultures, (c) IL-12p40 and IL-10 cytokine secretions were measured.

(d) Genotyping of TLR2- or MyD88-deficient mice was performed using qPCR with specific primers. These results are representative of at least three experiments.

Supplementary Figure 4

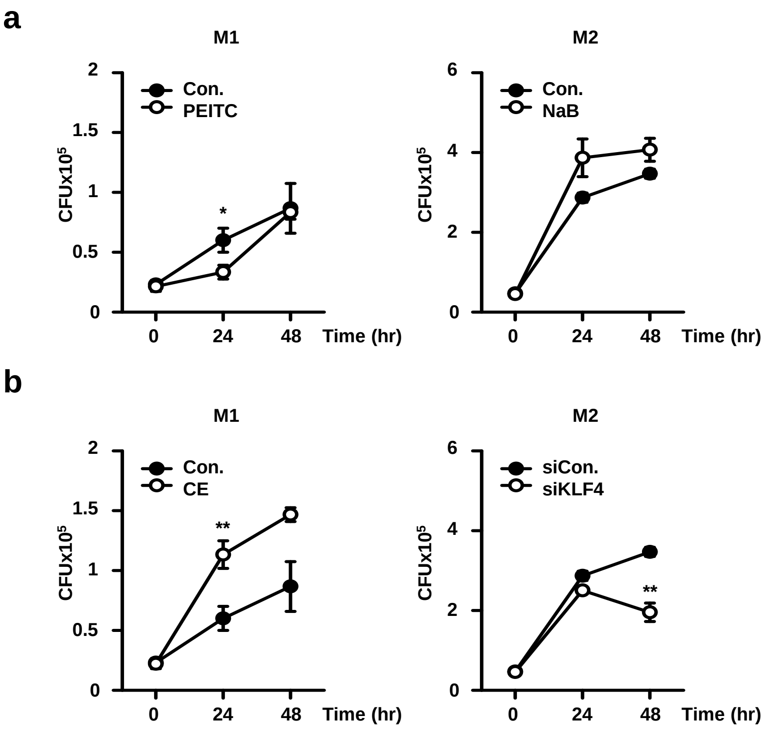

Supplementary figure 4, related to Figure 5. Macrophage polarization controls intracellular survival of H37Rv

(a) M1 and M2 macrophages were pre-treated with inducers for 2 h as follows: M1 macrophages received NICD inducer (PEITC, 2.5 μM) and M2 macrophages received KLF4 inducer (NaB, 1mM). M1 and M2 cells were then infected with H37Rv for 24 or 48 h. Intracellular survival of H37Rv was measured by CFU enumeration.

(b) M1 macrophages were pre-treated with γ-secretase inhibitor (compound E, 5 μM) for 2 h. M2 macrophages were transfected with KLF4 siRNA (200 nM) for 24 h. Then, M1 and M2 cells were infected with H37Rv for 24 or 48 h and analysed for the intracellular survival of Mtb.

Statistically significant differences are indicated; *p <0.05, ** p <0.01 and *** p <0.001. M0=unstimulated macrophages.

Supplementary Figure 5

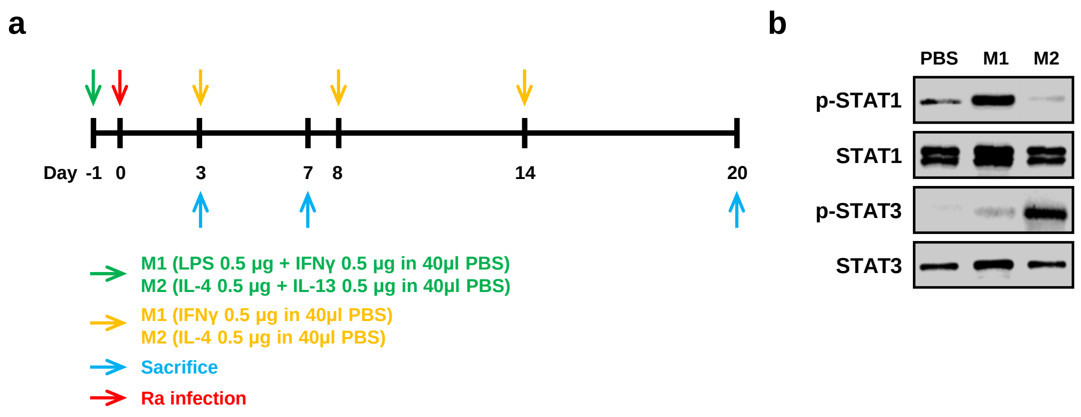

Supplementary figure 5, related to Figure 6a. Establishment of an in vivo mouse model to study macrophage polarization

(a) C57BL/6 mice were intranasally administered LPS and IFN-γ, or IL-4 and IL-13 as follows: For M1-like phenotype, 0.5 μg of LPS and 0.5 μg IFNγ in 40 μl PBS were administered; and for M2-lilke phenotype, 0.5 μg of IL-4 and 0.5 μg IL-13 in 40 μl PBS were administered according to the protocol, which was described in detail in method section.

(b) After administering the stimulants, the mice were infected with H37Ra ($5x10^6$) for 3 days. Whole-cell proteins were extracted by homogenizing the lungs in a lysis buffer. STAT1 and STAT3 phosphorylation was determined by western blot. Total STATs were used for cell loading controls. These results are representative of at least three independent experiments.

Supplementary Figure 6

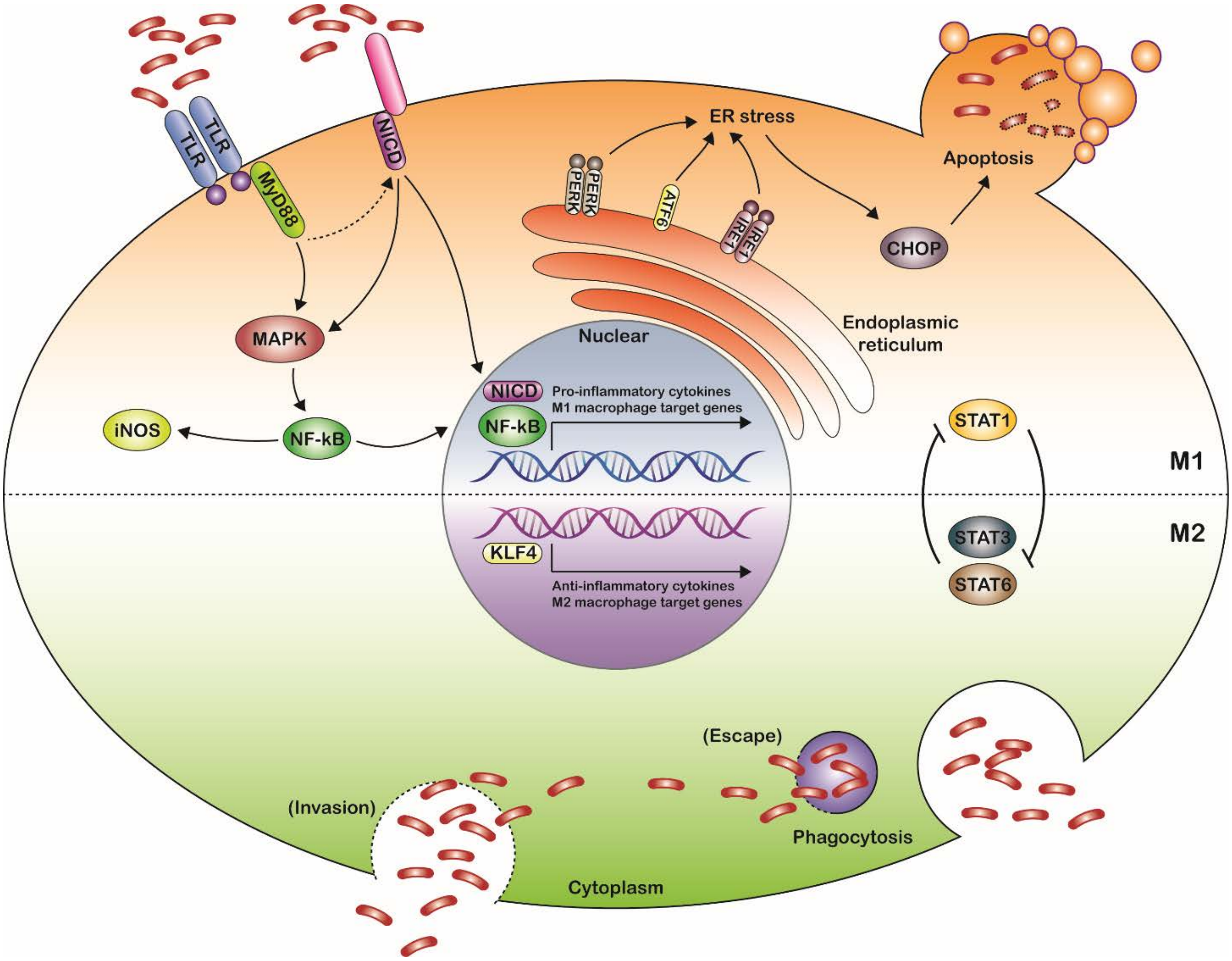

Supplementary figure 6, Schematic representation of macrophage polarization during mycobacterial infection

This illustration reflects that phenotypic and functional characteristics of macrophages are different in M1 *(upper)* and M2 *(lower)* macrophages during Mtb infection. M1 macrophages prove beneficial for removing intracellular survival of Mtb via ER stress-induced apoptotic pathway, in contrast, M2 macrophages appear as a favorable niche for long-term persistence of Mtb.
